# The crystal structure of bis­[(*E*)-4-bromo-2-({[2-(pyridin-2-yl)eth­yl]imino}­meth­yl)phenol]nickel(II) bis­[(*E*)-4-bromo-2-({[2-(pyridin-2-yl)eth­yl]imino}­meth­yl)phenolato]nickel(II) bis(perchlorate) methanol monosolvate, a structure containing strong inter-species hydrogen bonds

**DOI:** 10.1107/S2056989018010277

**Published:** 2018-07-20

**Authors:** Ugochukwu Okeke, Raymond Otchere, Yilma Gultneh, Ray J. Butcher

**Affiliations:** a Department of Chemistry, Howard University, 525 College Street NW, Washington, DC 20059, USA

**Keywords:** crystal structure, hydrogen bonding, nickel complexes, Schiff bases

## Abstract

The title compound, [Ni(C_14_H_12_BrN_2_O)_2_][Ni(C_14_H_13_BrN_2_O)_2_](ClO_4_)_2_·CH_3_OH consists of two mononuclear ([Ni(H*L*)_2_]^2+^ and [Ni*L*_2_]) complex mol­ecules linked by strong hydrogen bonding [O⋯O separations of only 2.430 (5) Å], which is the shortest reported to date for such species. In one of the complexes, both the coordinated phen­oxy groups retain their protons and thus this is the cationic equivalent species of the other complex where both coordinated phen­oxy groups are deprotonated. In addition, perchlorate anions are present for charge balance, as well as methanol solvate mol­ecules. For the neutral Ni*L*_2_ complex, each 2-ethyl­amine­pyridine arm is disordered over two equivalent conformations with occupancies of 0.750 (8):0.250 (8). The perchlorate anion is disordered over two equivalent conformations with occupancies of 0.602 (8):0.398 (8). The perchlorate ions also link to the H atoms on the methanol methyl and hydroxyl groups. These inter­actions link the moieties into a complex three-dimensional array. The crystal studied was refined as a two-component twin.

## Abstract 2

The title compound, [Ni(C_14_H_12_BrN_2_O)_2_][Ni(C_13_H_12_BrN_2_O)_2_](ClO_4_)_2_ 2(MeOH) consists of two mononuclear ([Ni(H*L*)_2_]^2+^ and [Ni*L*_2_]) complexes linked by strong hydrogen bonding [O⋯O separations of only 2.430 (5) Å], which is the shortest reported to date for such species.

## Chemical context   

Metal–Schiff base complexes have been of inter­est for a variety of reactions, in particular catalytic reactions (Egekenze *et al.*, 2017*a* ▸,*b* ▸, 2018*a* ▸,*b* ▸). The metalloenzyme urease contains Ni^II^ at its active site. Ureases can be found in a variety of species and efficiently accelerate by several orders of magnitude the rate of hydrolysis of urea into CO_2_ and NH_3_ (Mobley, 2001 ▸). It has been of great inter­est to catalyze a variety of reactions to mimic the catalytic efficiency of metalloenzymes. The crystal structures of related Ni^II^–Schiff base complexes have been reported (Ayikoé *et al.*, 2011 ▸; Butcher *et al.*, 2009 ▸; Elmali *et al.*, 2000 ▸; Kobayashi *et al.*, 2017 ▸; Kuchtanin *et al.*, 2016 ▸; Okeke *et al.*, 2017 ▸; Duran *et al.*, 1989 ▸). Similar complexes have been studied in relation to catalytic redox reactions, catechol oxidase activity, and alkaline phosphatase reactivity (Özalp-Yaman *et al.*, 2005 ▸; Sanyal *et al.*, 2016 ▸; Bhardwaj & Singh, 2014 ▸). In view of this inter­est and in a continuation of our previous research listed above, the title Ni^II^–Schiff base complex has been synthesized to be used as a catalyst for the hydrolysis of phosphate esters.
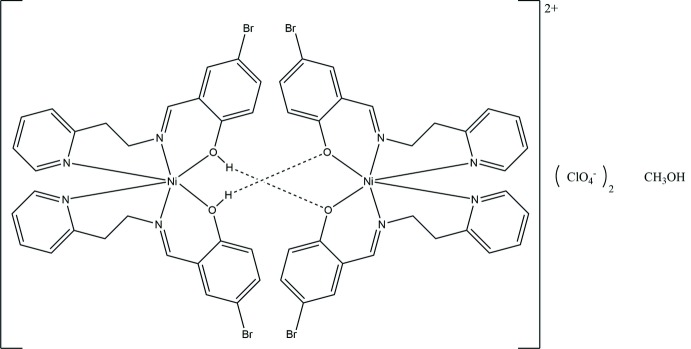


While the vast majority of such Ni complexes are of the type [Ni*L*_2_] where H*L* is the neutral Schiff base, there are a few examples where, upon coordination, the Schiff base retains its protons (You & Chi, 2006 ▸; Layek *et al.*, 2013 ▸; Ohta *et al.*, 2001 ▸; You *et al.*, 2004 ▸; Paital *et al.*, 2007 ▸; Xua *et al.*, 2015 ▸; Lucas *et al.*, 2011 ▸; Dutta *et al.*, 2010 ▸; Chakraborty *et al.*, 2006 ▸; Mukherjee *et al.*, 2007 ▸; Yamaguchi *et al.*, 2008 ▸; Fondo *et al.*, 2006 ▸; Zhang & Liang, 2017 ▸). The present structure is an unusual variant of this theme.

## Structural commentary   

**Figure 1:**
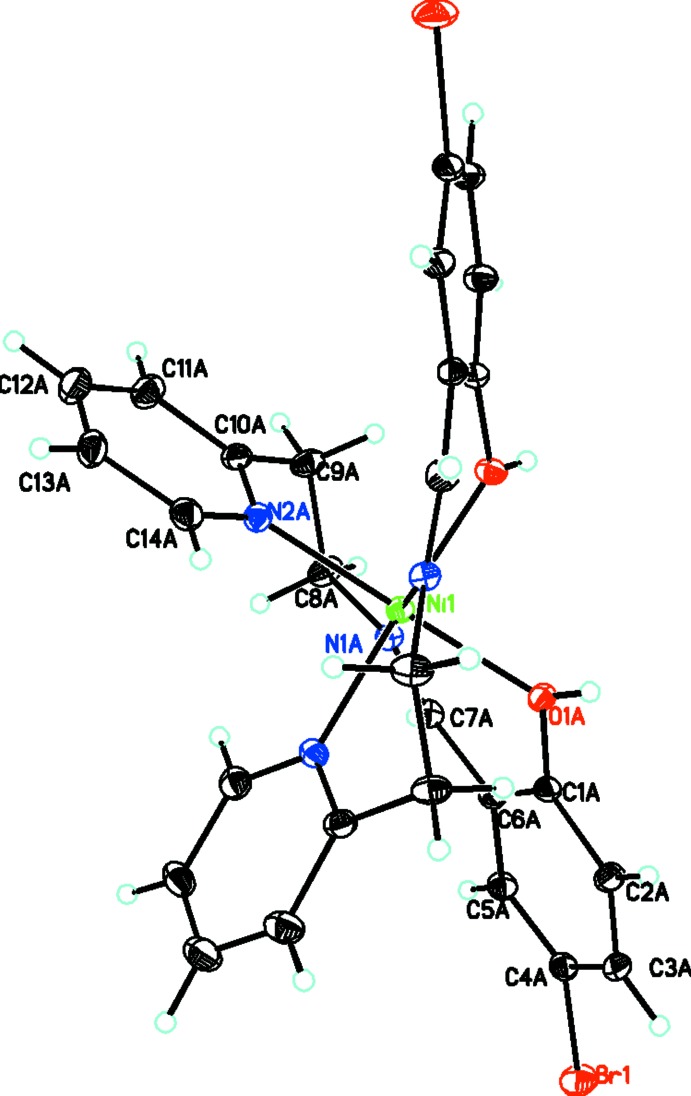
Diagram of the cation, {bis­[(*E*)-4-bromo-2-({[2-(pyridin-2-yl)eth­yl]imino}meth­yl)phenol]nickel(II)} showing the O—H phenol group coordinated to the nickel atom. Only the major component of the disordered group is shown. Atomic displacement parameters are at the 30% probability level. Unlabeled atoms are generated by the symmetry operation 1 − *x*, *y*, 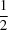 − *z*.

**Figure 3:**
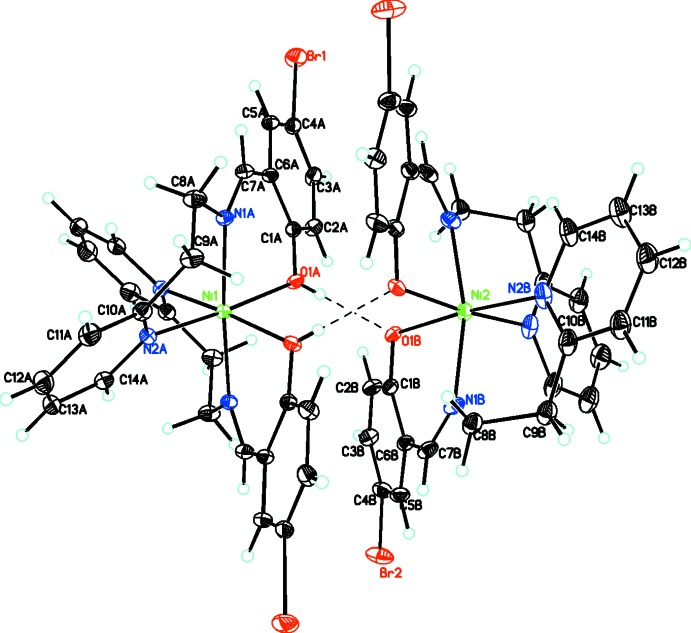
Diagram of both the cation and neutral complex linked by strong hydrogen bonding (shown as dashed lines). For the cation, only the major component of the disordered group is shown. Atomic displacement parameters are at the 30% probability level.

The title compound crystallizes in the ortho­rhom­bic space group *Pbcn* and consists of a coordination cation [Ni*L*_2_]^2+^, a neutral compound [Ni(H*L*_2_)] and perchlorate as anion to balance the charge. There is methanol in the lattice. Thus the stoichiometry is [Ni(H*L*)_2_]^2+^[Ni*L*_2_](ClO_4_^−^)_2_·MeOH. The Ni^II^ atoms are coordinated by nitro­gen and oxygen donor groups from the two tridentate ligands, thus making the Ni^II^ atoms six-coordinate (see Figs. 1 ▸ and 2 ▸). For the neutral Ni*L*_2_, the 2-ethyl­amine­pyridine arm is disordered over two equivalent conformations with occupancies of 0.750 (8):0.250 (8). The per­chlor­ate anion is disordered over two equivalent conformations with occupancies of 0.602 (8):0.398 (8). As noted in the synthesis section, no base was used in the preparation of the title compound, hence the presence of protonated (*i.e.* neutral) ligand mol­ecules. There is precedent in the literature (You & Chi, 2006 ▸; Layek *et al.*, 2013 ▸; Ohta *et al.*, 2001 ▸; You *et al.*, 2004 ▸; Paital *et al.*, 2007 ▸; Xua *et al.*, 2015 ▸; Lucas *et al.*, 2011 ▸; Dutta *et al.*, 2010 ▸; Chakraborty *et al.*, 2006 ▸; Mukherjee *et al.*, 2007 ▸; Yamaguchi *et al.*, 2008 ▸; Fondo *et al.*, 2006 ▸; Zhang & Liang, 2017 ▸) for nickel complexes with Schiff bases where the ligand is not deprotonated, although this is the only example where these are separated into independent metal complexes. A common motif of these examples is the presence of a strong inter­molecular hydrogen bond between these species with O⋯O separations ranging from 2.438 Å (Mukherjee *et al.*, 2007 ▸) to 2.592 Å (Layek, *et al.*, 2013 ▸). In the present case (Table 1 ▸, Fig. 3 ▸), this distance is 2.430 (5) Å, which is the shortest reported. The Ni^II^ atoms are coordinated to nitro­gen and oxygen donor groups from the two tridentate ligands, thus making the Ni^II^ atoms six-coordinate, with two perchlor­ate anions present for charge balance (see Fig. 1 ▸). While both Ni1 and Ni2 are six-coordinate, they are distorted from an octa­hedral geometry because of the chelate bite with *cis* angles ranging from 84.01 (16) to 93.07 (16)° for Ni1 and 84.10 (18) to 95.7 (6)° for Ni2. Surprisingly, the Ni—O bond lengths for Ni1 [2.070 (4) Å] are slightly shorter than for Ni2 [2.091 (4) Å], even though atom O1*A* is neutral and retains its proton while O1*B* is deprotonated and thus formally negatively charged. The Ni—N_imine_ and Ni—N_py_ bond lengths are 2.080 (4), 2.079 (5) Å and 2.095 (5), 2.128 (6) Å, respectively, with the bonds involving the imine group being shorter than those involving pyridine, as is expected based on the metrical parameters of similar complexes.

## Supra­molecular features   

**Figure 4:**
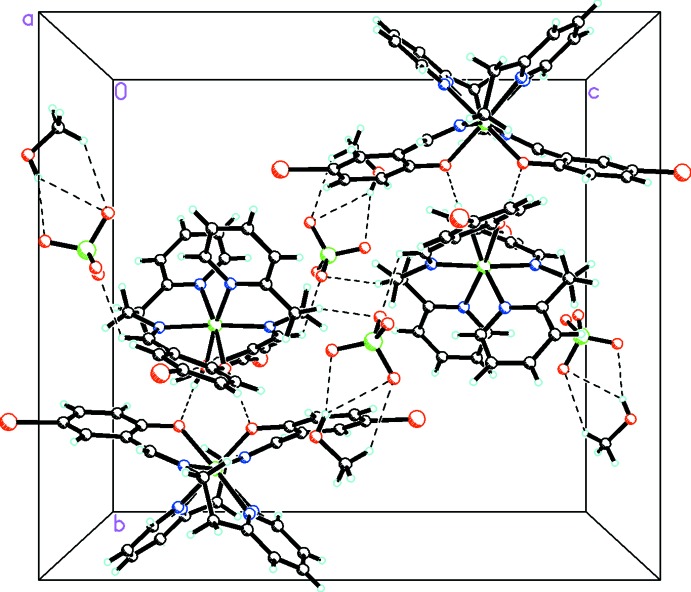
Packing diagram viewed along the *a* axis showing the extensive O—H⋯O, C—H⋯O, C—H⋯N, and C—H⋯Br inter­actions linking the cation, neutral complex, anion, and solvent mol­ecules into a three-dimensional array. For the disordered moieties, only the major conformation is shown.

The main point of inter­est in this structure is the presence of very strong inter-species hydrogen bonding between the phenol and phenolate moieties as mentioned above. In addition, the perchlorate anions link the complexes and methanol solvate mol­ecules through both C—H⋯O and O—H⋯O inter­actions (Table 1 ▸). These, along with C—H⋯Br inter­actions (Table 1 ▸), link all the species into a complex three-dimensional array as shown in Fig. 4 ▸.

## Database survey   

A search of the Cambridge Structural Database (CSD Version 5.39 with November 2017 update; Groom *et al.*, 2016 ▸) for similar Ni complexes of Schiff base ligands where the coord­inated O atoms are linked by O—H⋯O hydrogen bonds gave 15 hits (ADIKOO, You & Chi, 2006 ▸; HEWDUK, Layek *et al.*, 2013 ▸; IDAVOY, Ohta *et al.*, 2001 ▸; IWOVIZ, You *et al.*, 2004 ▸; LERXIS, Zhang & Liang, 2017 ▸; MIHJOD, Paital *et al.*, 2007 ▸; QUGZOJ, Xua *et al.*, 2015 ▸; UBICIT, Lucas *et al.*, 2011 ▸; UJUNIX, Dutta *et al.*, 2010 ▸; VESMAI, Chakraborty *et al.*, 2006 ▸; VIKMUY, Mukherjee *et al.*, 2007 ▸; WIZFAN, Yamaguchi *et al.*, 2008 ▸; YEQGIL, YEQHAE, YEQHEI, Fondo *et al.*, 2006 ▸).

## Synthesis and crystallization   

2-(2-Pyrid­yl)ethyl­amine (0.1613 g, 1.320 mmol) was added to a reaction flask and dissolved in 50 ml of methanol. 5-Bromo­salicyl­aldehyde (0.2654 g, 1.320 mmol) was added to the solution. The mixture was refluxed for 5 h. The nickel(II) complex was prepared by reacting the ligand in 50 ml of methanol with Ni(ClO_4_)_2_·6H_2_O (0.7242 g, 1.980 mmol) with no added base. The mixture was stirred at room temperature overnight. The product was crystallized by slow diffusion in methanol for two weeks giving green crystals.

## Refinement   

Crystal data, data collection and structure refinement details are summarized in Table 2 ▸. For the neutral NiL_2_, each 2-ethyl­amine­pyridine arm is disordered over two equivalent conformation with occupancies of 0.750 (8):0.250 (8). The perchlorate anion is disordered over two equivalent conformations with occupancies of 0.602 (8):0.398 (8). In addition there is pseudo-merohedral twinning present with a twin law of 0 0 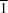 0 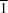 0 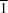 0 0 and BASF value of 0.0016 (3). The H atoms were positioned geometrically and allowed to ride on their parent atoms, with C—H ranging from 0.95 to 0.98 Å and *U*_iso_(H) = *xU*_eq_(C), where *x* = 1.5 for methyl H atoms and 1.2 for all other C-bound H atoms. The OH hydrogen atom was refined isotropically.

## Supplementary Material

Crystal structure: contains datablock(s) I. DOI: 10.1107/S2056989018010277/lh5876sup1.cif

Structure factors: contains datablock(s) I. DOI: 10.1107/S2056989018010277/lh5876Isup2.hkl

CCDC reference: 1856226

Additional supporting information:  crystallographic information; 3D view; checkCIF report

## Figures and Tables

**Figure 2:**
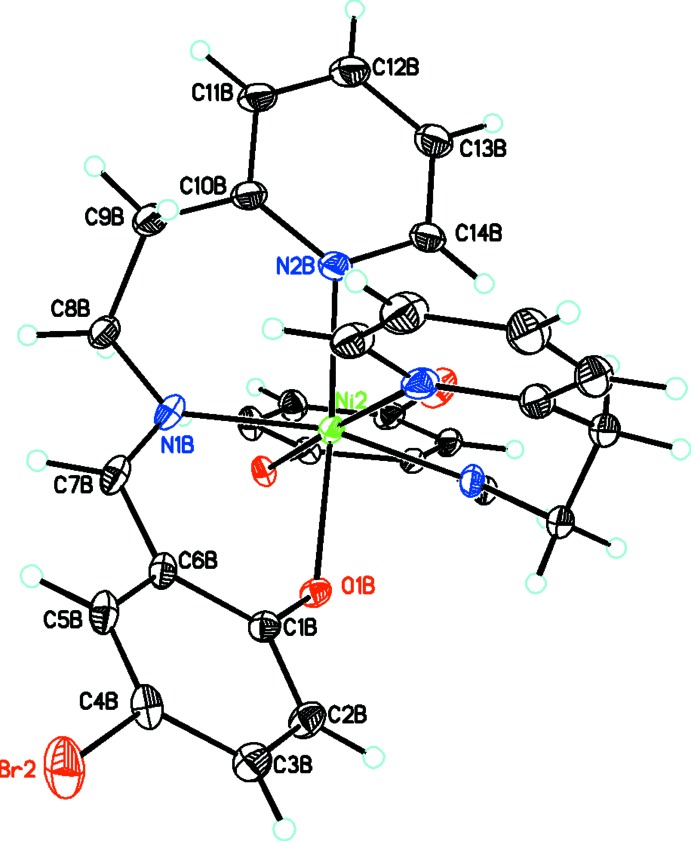
Diagram of the neutral complex, {bis­[(*E*)-4-bromo-2-({[2-(pyridin-2-yl)eth­yl]imino}­meth­yl)phenolato]nickel(II)}. Atomic displacement parameters are at the 30% probability level. Unlabeled atoms are generated by the symmetry operation 1 − *x*, *y*, 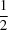 − *z*.

**Table 1:** Hydrogen-bond geometry (Å, °)

*D*—H⋯*A*	*D*—H	H⋯*A*	*D*⋯*A*	*D*—H⋯*A*
O1*A*—H1*A*⋯O1*B*	0.82 (2)	1.64 (3)	2.430 (5)	161 (8)
C9*A*—H9*AA*⋯O1*A* ^i^	0.97	2.40	3.105 (7)	129
C9*A*—H9*AB*⋯O14^ii^	0.97	2.55	3.482 (12)	162
C11*A*—H11*A*⋯O14*A* ^ii^	0.93	2.60	3.406 (12)	145
C9*B*—H9*BB*⋯Br1^iii^	0.97	3.12	3.859 (10)	134
C14*B*—H14*B*⋯N1*B* ^i^	0.93	2.54	3.155 (9)	124
C9*C*—H9*CA*⋯O1*B* ^i^	0.97	2.37	3.02 (3)	124
O1*S*—H1*S*⋯O12	0.82	2.12	2.907 (15)	162
O1*S*—H1*S*⋯O13	0.82	2.57	3.249 (15)	140
O1*S*—H1*S*⋯O13*A*	0.82	1.64	2.436 (16)	162
C1*S*—H1*S*3⋯O13	0.96	2.55	3.276 (19)	133

Symmetry codes: (i) 
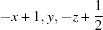
; (ii) 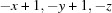; (iii) 
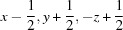
.

**Table 2:** Experimental details

Crystal data	
Chemical formula	[Ni(C_14_H_12_BrN_2_O)_2_][Ni(C_14_H_13_BrN_2_O)_2_](ClO_4_)_2_·CH_4_O
*M*_r_	1567.04
Crystal system, space group	Orthorhombic, *P* *b* *c* *n*
Temperature (K)	296
*a*, *b*, *c* (Å)	19.103 (5), 17.414 (4), 19.053 (5)
*V* (Å^3^)	6339 (3)
*Z*	4
Radiation type	Mo *K*α
μ (mm^−1^)	3.27
Crystal size (mm)	0.32 × 0.28 × 0.13

Data collection	
Diffractometer	Bruker APEXII CCD
Absorption correction	Multi-scan (*SADABS*; Sheldrick, 1996 ▸)
*T*_min_, *T*_max_	0.433, 0.745
No. of measured, independent and observed [*I* > 2σ(*I*)] reflections	6170, 6170, 3693
*R*_int_	0.088
(sin θ/λ)_max_ (Å^−1^)	0.629

Refinement	
*R*[*F* ^2^ > 2σ(*F* ^2^)], *wR*(*F* ^2^), *S*	0.061, 0.178, 1.02
No. of reflections	6170
No. of parameters	492
No. of restraints	332
H-atom treatment	H atoms treated by a mixture of independent and constrained refinement
Δρ_max_, Δρ_min_ (e Å^−3^)	0.90, −0.89

Computer programs: *APEX3* and *SAINT* (Bruker, 2012 ▸), *SHELXT2014/5* (Sheldrick, 2015*a* ▸), *SHELXL2018/1* (Sheldrick, 2015*b* ▸) and *SHELXTL* (Sheldrick, 2008 ▸).

## supplementary crystallographic information

### Crystal data

**Table:**

[Ni(C_14_H_12_BrN_2_O)_2_][Ni(C_14_H_13_BrN_2_O)_2_](ClO_4_)_2_·CH_4_O	*D*_x_ = 1.642 Mg m^−^^3^
*M**_r_* = 1567.04	Mo *K*α radiation, λ = 0.71073 Å
Orthorhombic, *P**b**c**n*	Cell parameters from 4627 reflections
*a* = 19.103 (5) Å	θ = 2.4–26.3°
*b* = 17.414 (4) Å	µ = 3.27 mm^−^^1^
*c* = 19.053 (5) Å	*T* = 296 K
*V* = 6339 (3) Å^3^	Prism, transparent light olive-green
*Z* = 4	0.32 × 0.28 × 0.13 mm
*F*(000) = 3144	

### Data collection

**Table:**

Bruker APEXII CCD diffractometer	3693 reflections with *I* > 2σ(*I*)
w scans	*R*_int_ = 0.088
Absorption correction: multi-scan (SADABS; Sheldrick, 1996)	θ_max_ = 26.5°, θ_min_ = 1.6°
*T*_min_ = 0.433, *T*_max_ = 0.745	*h* = −16→23
6170 measured reflections	*k* = −17→19
6170 independent reflections	*l* = −22→23

### Refinement

**Table:**

Refinement on *F*^2^	332 restraints
Least-squares matrix: full	Hydrogen site location: mixed
*R*[*F*^2^ > 2σ(*F*^2^)] = 0.061	H atoms treated by a mixture of independent and constrained refinement
*wR*(*F*^2^) = 0.178	*w* = 1/[σ^2^(*F*_o_^2^) + (0.0767*P*)^2^ + 9.3718*P*] where *P* = (*F*_o_^2^ + 2*F*_c_^2^)/3
*S* = 1.01	(Δ/σ)_max_ < 0.001
6170 reflections	Δρ_max_ = 0.90 e Å^−^^3^
492 parameters	Δρ_min_ = −0.89 e Å^−^^3^

### Special details

**Table:**

Geometry. All esds (except the esd in the dihedral angle between two l.s. planes) are estimated using the full covariance matrix. The cell esds are taken into account individually in the estimation of esds in distances, angles and torsion angles; correlations between esds in cell parameters are only used when they are defined by crystal symmetry. An approximate (isotropic) treatment of cell esds is used for estimating esds involving l.s. planes.
Refinement. Refined as a two-component twin.

### Fractional atomic coordinates and isotropic or equivalent isotropic displacement parameters (Å^2^)

**Table:**

	*x*	*y*	*z*	*U*_iso_*/*U*_eq_	Occ. (<1)
Ni1	0.500000	0.55970 (5)	0.250000	0.0426 (3)	
Br1	0.87543 (3)	0.64179 (5)	0.18413 (5)	0.0946 (3)	
O1A	0.57178 (18)	0.6453 (2)	0.2724 (2)	0.0503 (9)	
H1A	0.567 (4)	0.686 (3)	0.294 (4)	0.11 (3)*	
N1A	0.5439 (2)	0.5639 (3)	0.1502 (2)	0.0466 (11)	
N2A	0.4284 (2)	0.4763 (3)	0.2165 (2)	0.0502 (11)	
C1A	0.6398 (3)	0.6417 (3)	0.2536 (3)	0.0478 (12)	
C2A	0.6928 (3)	0.6663 (4)	0.2984 (3)	0.0607 (16)	
H2AA	0.681286	0.683694	0.343027	0.073*	
C3A	0.7625 (3)	0.6653 (4)	0.2778 (4)	0.0636 (17)	
H3AA	0.797194	0.682336	0.308245	0.076*	
C4A	0.7794 (3)	0.6394 (3)	0.2132 (4)	0.0608 (16)	
C5A	0.7296 (3)	0.6124 (3)	0.1678 (3)	0.0581 (15)	
H5AA	0.742734	0.593314	0.124161	0.070*	
C6A	0.6583 (2)	0.6133 (3)	0.1872 (3)	0.0463 (13)	
C7A	0.6069 (3)	0.5853 (3)	0.1373 (3)	0.0532 (14)	
H7AA	0.621250	0.582503	0.090759	0.064*	
C8A	0.5050 (3)	0.5342 (4)	0.0888 (3)	0.0662 (17)	
H8AA	0.520105	0.482112	0.079184	0.079*	
H8AB	0.516229	0.565325	0.048055	0.079*	
C9A	0.4255 (3)	0.5347 (4)	0.0998 (3)	0.0635 (17)	
H9AA	0.411465	0.585451	0.115535	0.076*	
H9AB	0.402788	0.525256	0.055118	0.076*	
C10A	0.4001 (3)	0.4772 (4)	0.1514 (3)	0.0562 (15)	
C11A	0.3483 (4)	0.4241 (4)	0.1335 (4)	0.077 (2)	
H11A	0.328428	0.425746	0.088979	0.093*	
C12A	0.3262 (4)	0.3696 (5)	0.1806 (5)	0.088 (2)	
H12A	0.291893	0.334153	0.168416	0.105*	
C13A	0.3560 (4)	0.3686 (4)	0.2461 (4)	0.077 (2)	
H13A	0.342541	0.332136	0.279138	0.092*	
C14A	0.4063 (3)	0.4225 (4)	0.2620 (3)	0.0647 (17)	
H14A	0.426067	0.421569	0.306602	0.078*	
Ni2	0.500000	0.85234 (6)	0.250000	0.0536 (3)	
Br2	0.45642 (5)	0.75407 (7)	0.62684 (4)	0.1140 (4)	
O1B	0.53437 (18)	0.7680 (2)	0.32016 (19)	0.0532 (9)	
N1B	0.4042 (2)	0.8375 (3)	0.2999 (3)	0.0628 (14)	
C1B	0.5161 (3)	0.7651 (3)	0.3887 (3)	0.0532 (14)	
C2B	0.5638 (3)	0.7434 (4)	0.4396 (3)	0.0689 (18)	
H2BA	0.609303	0.731075	0.426250	0.083*	
C3B	0.5457 (4)	0.7395 (4)	0.5095 (4)	0.0718 (18)	
H3BA	0.578777	0.725434	0.542938	0.086*	
C4B	0.4787 (3)	0.7565 (4)	0.5292 (3)	0.0694 (19)	
C5B	0.4306 (3)	0.7773 (4)	0.4815 (4)	0.0682 (18)	
H5BA	0.385214	0.788122	0.496166	0.082*	
C6B	0.4474 (3)	0.7833 (3)	0.4096 (3)	0.0577 (15)	
C7B	0.3934 (3)	0.8077 (4)	0.3602 (4)	0.0672 (18)	
H7BA	0.347101	0.800911	0.373764	0.081*	
C8B	0.3418 (7)	0.8540 (6)	0.2606 (6)	0.072 (3)	0.750 (8)
H8BA	0.300765	0.841264	0.288191	0.087*	0.750 (8)
H8BB	0.340919	0.823569	0.217958	0.087*	0.750 (8)
C9B	0.3412 (5)	0.9387 (5)	0.2424 (5)	0.078 (2)	0.750 (8)
H9BA	0.293708	0.953252	0.230515	0.094*	0.750 (8)
H9BB	0.354464	0.967433	0.283973	0.094*	0.750 (8)
N2B	0.4574 (3)	0.9393 (4)	0.1842 (4)	0.0778 (18)	0.750 (8)
C10B	0.3876 (3)	0.9618 (4)	0.1844 (4)	0.081 (2)	0.750 (8)
C11B	0.3634 (3)	1.0139 (4)	0.1347 (5)	0.094 (2)	0.750 (8)
H11B	0.316734	1.028997	0.134862	0.113*	0.750 (8)
C12B	0.4091 (4)	1.0436 (4)	0.0848 (4)	0.104 (3)	0.750 (8)
H12B	0.393009	1.078435	0.051579	0.125*	0.750 (8)
C13B	0.4790 (4)	1.0211 (5)	0.0846 (4)	0.104 (3)	0.750 (8)
H13B	0.509529	1.040883	0.051222	0.125*	0.750 (8)
C14B	0.5031 (3)	0.9689 (5)	0.1343 (4)	0.087 (2)	0.750 (8)
H14B	0.549774	0.953894	0.134149	0.105*	0.750 (8)
C8C	0.340 (2)	0.877 (3)	0.2588 (15)	0.075 (4)	0.250 (8)
H8CA	0.298029	0.848864	0.270933	0.090*	0.250 (8)
H8CB	0.335150	0.928550	0.276206	0.090*	0.250 (8)
C9C	0.3451 (12)	0.8802 (13)	0.1796 (12)	0.078 (3)	0.250 (8)
H9CA	0.356268	0.829295	0.162178	0.094*	0.250 (8)
H9CB	0.299344	0.893832	0.161295	0.094*	0.250 (8)
N2C	0.4645 (9)	0.9346 (14)	0.1779 (12)	0.082 (3)	0.250 (8)
C10C	0.3968 (8)	0.9346 (12)	0.1512 (12)	0.086 (3)	0.250 (8)
C11C	0.3795 (10)	0.9826 (13)	0.0956 (13)	0.094 (3)	0.250 (8)
H11C	0.334160	0.982612	0.077782	0.112*	0.250 (8)
C12C	0.4299 (13)	1.0306 (13)	0.0666 (12)	0.101 (3)	0.250 (8)
H12C	0.418312	1.062737	0.029362	0.121*	0.250 (8)
C13C	0.4976 (12)	1.0306 (15)	0.0932 (15)	0.099 (3)	0.250 (8)
H13C	0.531373	1.062758	0.073817	0.119*	0.250 (8)
C14C	0.5150 (9)	0.9826 (16)	0.1489 (15)	0.090 (3)	0.250 (8)
H14C	0.560283	0.982653	0.166693	0.109*	0.250 (8)
Cl1	0.73020 (12)	0.41261 (15)	0.04156 (12)	0.1002 (7)	
O11	0.7917 (4)	0.4471 (5)	0.0653 (5)	0.145 (4)	0.602 (8)
O12	0.7358 (6)	0.3960 (6)	−0.0303 (3)	0.135 (4)	0.602 (8)
O13	0.7219 (5)	0.3417 (5)	0.0779 (5)	0.145 (4)	0.602 (8)
O14	0.6721 (5)	0.4583 (6)	0.0544 (6)	0.179 (5)	0.602 (8)
O11A	0.7786 (8)	0.4212 (8)	0.0960 (7)	0.157 (6)	0.398 (8)
O12A	0.6654 (5)	0.3894 (10)	0.0683 (9)	0.175 (6)	0.398 (8)
O13A	0.7546 (9)	0.3604 (8)	−0.0080 (7)	0.156 (6)	0.398 (8)
O14A	0.7209 (8)	0.4854 (5)	0.0090 (7)	0.133 (5)	0.398 (8)
O1S	0.7449 (6)	0.2322 (7)	−0.0582 (6)	0.094 (3)	0.5
H1S	0.739358	0.275191	−0.041527	0.141*	0.5
C1S	0.7385 (9)	0.1792 (10)	−0.0070 (8)	0.098 (5)	0.5
H1S1	0.783439	0.156617	0.002282	0.146*	0.5
H1S2	0.706437	0.139927	−0.021727	0.146*	0.5
H1S3	0.721208	0.203222	0.034875	0.146*	0.5

### Atomic displacement parameters (Å^2^)

**Table:**

	*U*^11^	*U*^22^	*U*^33^	*U*^12^	*U*^13^	*U*^23^
Ni1	0.0392 (5)	0.0488 (6)	0.0399 (5)	0.000	0.0026 (4)	0.000
Br1	0.0406 (3)	0.1081 (7)	0.1351 (7)	−0.0032 (4)	0.0209 (4)	−0.0139 (5)
O1A	0.0375 (18)	0.055 (3)	0.058 (2)	−0.0020 (18)	0.0072 (16)	−0.018 (2)
N1A	0.046 (2)	0.054 (3)	0.039 (2)	0.001 (2)	0.0004 (18)	−0.002 (2)
N2A	0.049 (2)	0.049 (3)	0.053 (3)	−0.004 (2)	0.003 (2)	0.000 (2)
C1A	0.038 (3)	0.046 (3)	0.060 (3)	0.001 (2)	0.003 (2)	0.002 (3)
C2A	0.048 (3)	0.067 (4)	0.067 (4)	−0.002 (3)	−0.003 (3)	−0.016 (3)
C3A	0.044 (3)	0.058 (4)	0.089 (5)	0.000 (3)	−0.006 (3)	−0.017 (3)
C4A	0.039 (3)	0.052 (4)	0.092 (5)	0.002 (3)	0.015 (3)	−0.004 (3)
C5A	0.044 (3)	0.060 (4)	0.071 (4)	0.003 (3)	0.018 (3)	−0.004 (3)
C6A	0.038 (3)	0.051 (3)	0.050 (3)	0.003 (2)	0.007 (2)	0.000 (3)
C7A	0.052 (3)	0.064 (4)	0.043 (3)	0.003 (3)	0.010 (2)	−0.001 (3)
C8A	0.059 (3)	0.095 (5)	0.044 (3)	−0.010 (3)	0.000 (3)	−0.014 (3)
C9A	0.050 (3)	0.091 (5)	0.050 (3)	−0.004 (3)	−0.009 (3)	−0.013 (3)
C10A	0.044 (3)	0.062 (4)	0.063 (4)	0.003 (3)	−0.003 (3)	−0.006 (3)
C11A	0.068 (4)	0.084 (5)	0.080 (5)	−0.010 (4)	−0.004 (4)	−0.022 (4)
C12A	0.074 (5)	0.077 (6)	0.113 (7)	−0.017 (4)	0.003 (4)	−0.020 (5)
C13A	0.070 (4)	0.056 (4)	0.104 (6)	−0.013 (3)	0.024 (4)	−0.006 (4)
C14A	0.059 (4)	0.062 (4)	0.073 (4)	0.005 (3)	0.012 (3)	−0.003 (3)
Ni2	0.0385 (5)	0.0453 (6)	0.0770 (7)	0.000	−0.0050 (5)	0.000
Br2	0.0980 (6)	0.1772 (11)	0.0669 (5)	−0.0334 (6)	0.0250 (4)	0.0002 (5)
O1B	0.051 (2)	0.053 (2)	0.055 (2)	0.0138 (18)	0.0065 (17)	−0.0076 (18)
N1B	0.039 (2)	0.079 (4)	0.070 (3)	0.009 (2)	−0.001 (2)	−0.016 (3)
C1B	0.054 (3)	0.049 (4)	0.057 (3)	0.005 (3)	0.005 (3)	−0.008 (3)
C2B	0.060 (4)	0.082 (5)	0.065 (4)	0.018 (3)	0.009 (3)	−0.006 (3)
C3B	0.072 (4)	0.078 (5)	0.066 (4)	0.007 (4)	0.003 (3)	−0.007 (4)
C4B	0.065 (4)	0.082 (5)	0.060 (4)	−0.024 (4)	0.011 (3)	−0.010 (3)
C5B	0.051 (3)	0.077 (5)	0.077 (4)	−0.012 (3)	0.019 (3)	−0.018 (4)
C6B	0.044 (3)	0.056 (4)	0.074 (4)	−0.006 (3)	0.010 (3)	−0.016 (3)
C7B	0.038 (3)	0.076 (5)	0.087 (5)	0.002 (3)	0.005 (3)	−0.030 (4)
C8B	0.048 (4)	0.066 (6)	0.103 (5)	0.007 (5)	−0.007 (4)	−0.011 (4)
C9B	0.058 (4)	0.069 (5)	0.108 (5)	0.008 (4)	−0.015 (4)	−0.007 (4)
N2B	0.070 (4)	0.052 (4)	0.111 (4)	−0.006 (3)	−0.028 (3)	0.011 (3)
C10B	0.071 (4)	0.058 (4)	0.114 (4)	0.007 (3)	−0.025 (4)	0.008 (4)
C11B	0.083 (5)	0.071 (5)	0.128 (5)	0.015 (4)	−0.026 (4)	0.019 (4)
C12B	0.098 (5)	0.081 (5)	0.133 (5)	0.014 (4)	−0.029 (4)	0.028 (4)
C13B	0.101 (5)	0.080 (5)	0.132 (5)	−0.009 (4)	−0.030 (5)	0.028 (4)
C14B	0.083 (4)	0.062 (4)	0.117 (5)	−0.008 (4)	−0.035 (4)	0.023 (4)
C8C	0.052 (6)	0.067 (8)	0.105 (6)	0.010 (7)	−0.013 (6)	−0.009 (7)
C9C	0.059 (5)	0.066 (6)	0.109 (5)	0.010 (5)	−0.017 (5)	−0.002 (5)
N2C	0.074 (5)	0.057 (5)	0.114 (5)	−0.002 (5)	−0.029 (5)	0.012 (5)
C10C	0.075 (5)	0.064 (5)	0.118 (5)	0.006 (5)	−0.023 (5)	0.011 (5)
C11C	0.084 (5)	0.072 (5)	0.126 (6)	0.010 (5)	−0.027 (5)	0.018 (5)
C12C	0.093 (6)	0.078 (5)	0.132 (6)	0.009 (5)	−0.028 (5)	0.026 (5)
C13C	0.095 (6)	0.074 (5)	0.128 (6)	−0.003 (5)	−0.033 (5)	0.025 (5)
C14C	0.087 (5)	0.064 (5)	0.120 (6)	−0.007 (5)	−0.031 (5)	0.020 (5)
Cl1	0.0979 (15)	0.1148 (19)	0.0879 (14)	0.0206 (14)	−0.0121 (11)	−0.0020 (13)
O11	0.159 (9)	0.137 (9)	0.141 (9)	−0.016 (7)	−0.045 (8)	0.059 (7)
O12	0.181 (10)	0.139 (10)	0.085 (7)	0.047 (8)	0.013 (7)	−0.004 (6)
O13	0.103 (7)	0.175 (10)	0.158 (8)	−0.006 (7)	−0.017 (7)	0.044 (8)
O14	0.167 (10)	0.213 (12)	0.157 (10)	0.109 (10)	−0.025 (8)	−0.031 (10)
O11A	0.169 (11)	0.147 (12)	0.154 (12)	−0.003 (10)	−0.093 (10)	0.062 (10)
O12A	0.156 (12)	0.200 (15)	0.168 (12)	0.000 (12)	0.051 (11)	0.028 (13)
O13A	0.175 (12)	0.157 (13)	0.137 (12)	0.063 (11)	0.015 (11)	−0.008 (11)
O14A	0.141 (10)	0.149 (10)	0.108 (9)	0.035 (9)	−0.020 (8)	0.034 (9)
O1S	0.091 (7)	0.098 (8)	0.093 (7)	0.033 (7)	0.005 (6)	−0.012 (7)
C1S	0.120 (11)	0.089 (10)	0.084 (9)	0.068 (9)	0.015 (8)	0.019 (8)

### Geometric parameters (Å, º)

**Table:**

Ni1—O1A	2.070 (4)	C2B—H2BA	0.9300
Ni1—O1A^i^	2.070 (4)	C3B—C4B	1.367 (9)
Ni1—N1A	2.080 (4)	C3B—H3BA	0.9300
Ni1—N1A^i^	2.080 (4)	C4B—C5B	1.342 (9)
Ni1—N2A	2.095 (5)	C5B—C6B	1.411 (9)
Ni1—N2A^i^	2.095 (5)	C5B—H5BA	0.9300
Br1—C4A	1.917 (5)	C6B—C7B	1.459 (9)
O1A—C1A	1.349 (6)	C7B—H7BA	0.9300
O1A—H1A	0.82 (2)	C8B—C9B	1.514 (14)
N1A—C7A	1.283 (6)	C8B—H8BA	0.9700
N1A—C8A	1.480 (7)	C8B—H8BB	0.9700
N2A—C14A	1.344 (7)	C9B—C10B	1.472 (11)
N2A—C10A	1.353 (7)	C9B—H9BA	0.9700
C1A—C2A	1.391 (8)	C9B—H9BB	0.9700
C1A—C6A	1.404 (7)	N2B—C10B	1.3900
C2A—C3A	1.387 (8)	N2B—C14B	1.3900
C2A—H2AA	0.9300	C10B—C11B	1.3900
C3A—C4A	1.350 (9)	C11B—C12B	1.3900
C3A—H3AA	0.9300	C11B—H11B	0.9300
C4A—C5A	1.370 (9)	C12B—C13B	1.3900
C5A—C6A	1.411 (7)	C12B—H12B	0.9300
C5A—H5AA	0.9300	C13B—C14B	1.3900
C6A—C7A	1.451 (7)	C13B—H13B	0.9300
C7A—H7AA	0.9300	C14B—H14B	0.9300
C8A—C9A	1.534 (8)	C8C—C9C	1.513 (15)
C8A—H8AA	0.9700	C8C—H8CA	0.9700
C8A—H8AB	0.9700	C8C—H8CB	0.9700
C9A—C10A	1.485 (9)	C9C—C10C	1.472 (11)
C9A—H9AA	0.9700	C9C—H9CA	0.9700
C9A—H9AB	0.9700	C9C—H9CB	0.9700
C10A—C11A	1.396 (9)	N2C—C10C	1.3900
C11A—C12A	1.373 (10)	N2C—C14C	1.3900
C11A—H11A	0.9300	C10C—C11C	1.3900
C12A—C13A	1.371 (11)	C11C—C12C	1.3900
C12A—H12A	0.9300	C11C—H11C	0.9300
C13A—C14A	1.377 (9)	C12C—C13C	1.3900
C13A—H13A	0.9300	C12C—H12C	0.9300
C14A—H14A	0.9300	C13C—C14C	1.3900
Ni2—N1B	2.079 (5)	C13C—H13C	0.9300
Ni2—N1B^i^	2.079 (5)	C14C—H14C	0.9300
Ni2—O1B^i^	2.091 (4)	Cl1—O14	1.387 (6)
Ni2—O1B	2.091 (4)	Cl1—O13A	1.391 (6)
Ni2—N2C	2.098 (18)	Cl1—O11	1.396 (6)
Ni2—N2C^i^	2.098 (18)	Cl1—O11A	1.397 (6)
Ni2—N2B^i^	2.128 (6)	Cl1—O12A	1.399 (6)
Ni2—N2B	2.128 (6)	Cl1—O12	1.403 (6)
Br2—C4B	1.909 (6)	Cl1—O14A	1.422 (6)
O1B—C1B	1.353 (7)	Cl1—O13	1.424 (6)
N1B—C7B	1.277 (8)	O1S—C1S	1.349 (17)
N1B—C8B	1.436 (14)	O1S—H1S	0.8200
N1B—C8C	1.60 (5)	C1S—H1S1	0.9600
C1B—C2B	1.383 (9)	C1S—H1S2	0.9600
C1B—C6B	1.409 (8)	C1S—H1S3	0.9600
C2B—C3B	1.378 (9)		
			
O1A—Ni1—O1A^i^	87.8 (2)	C8C—N1B—Ni2	113.2 (10)
O1A—Ni1—N1A	84.01 (16)	O1B—C1B—C2B	121.2 (5)
O1A^i^—Ni1—N1A	93.07 (16)	O1B—C1B—C6B	120.2 (5)
O1A—Ni1—N1A^i^	93.07 (16)	C2B—C1B—C6B	118.6 (6)
O1A^i^—Ni1—N1A^i^	84.01 (16)	C3B—C2B—C1B	121.7 (6)
N1A—Ni1—N1A^i^	176.0 (2)	C3B—C2B—H2BA	119.2
O1A—Ni1—N2A	174.13 (17)	C1B—C2B—H2BA	119.2
O1A^i^—Ni1—N2A	90.23 (17)	C4B—C3B—C2B	119.3 (7)
N1A—Ni1—N2A	90.56 (17)	C4B—C3B—H3BA	120.3
N1A^i^—Ni1—N2A	92.25 (17)	C2B—C3B—H3BA	120.3
O1A—Ni1—N2A^i^	90.23 (17)	C5B—C4B—C3B	120.9 (6)
O1A^i^—Ni1—N2A^i^	174.13 (17)	C5B—C4B—Br2	120.9 (5)
N1A—Ni1—N2A^i^	92.25 (17)	C3B—C4B—Br2	118.1 (5)
N1A^i^—Ni1—N2A^i^	90.56 (17)	C4B—C5B—C6B	121.5 (6)
N2A—Ni1—N2A^i^	92.2 (3)	C4B—C5B—H5BA	119.3
C1A—O1A—Ni1	123.4 (3)	C6B—C5B—H5BA	119.3
C1A—O1A—H1A	107 (6)	C1B—C6B—C5B	118.0 (6)
Ni1—O1A—H1A	130 (6)	C1B—C6B—C7B	122.8 (6)
C7A—N1A—C8A	115.0 (4)	C5B—C6B—C7B	119.2 (5)
C7A—N1A—Ni1	124.3 (4)	N1B—C7B—C6B	125.8 (5)
C8A—N1A—Ni1	120.5 (3)	N1B—C7B—H7BA	117.1
C14A—N2A—C10A	118.3 (5)	C6B—C7B—H7BA	117.1
C14A—N2A—Ni1	119.5 (4)	N1B—C8B—C9B	108.6 (9)
C10A—N2A—Ni1	122.1 (4)	N1B—C8B—H8BA	110.0
O1A—C1A—C2A	121.6 (5)	C9B—C8B—H8BA	110.0
O1A—C1A—C6A	119.9 (5)	N1B—C8B—H8BB	110.0
C2A—C1A—C6A	118.5 (5)	C9B—C8B—H8BB	110.0
C3A—C2A—C1A	121.4 (6)	H8BA—C8B—H8BB	108.3
C3A—C2A—H2AA	119.3	C10B—C9B—C8B	115.7 (8)
C1A—C2A—H2AA	119.3	C10B—C9B—H9BA	108.4
C4A—C3A—C2A	119.4 (6)	C8B—C9B—H9BA	108.4
C4A—C3A—H3AA	120.3	C10B—C9B—H9BB	108.4
C2A—C3A—H3AA	120.3	C8B—C9B—H9BB	108.4
C3A—C4A—C5A	121.6 (5)	H9BA—C9B—H9BB	107.4
C3A—C4A—Br1	119.1 (5)	C10B—N2B—C14B	120.0
C5A—C4A—Br1	119.3 (5)	C10B—N2B—Ni2	124.5 (3)
C4A—C5A—C6A	120.1 (5)	C14B—N2B—Ni2	115.3 (3)
C4A—C5A—H5AA	120.0	C11B—C10B—N2B	120.0
C6A—C5A—H5AA	120.0	C11B—C10B—C9B	119.4 (5)
C1A—C6A—C5A	118.9 (5)	N2B—C10B—C9B	120.1 (5)
C1A—C6A—C7A	122.6 (4)	C10B—C11B—C12B	120.0
C5A—C6A—C7A	118.5 (5)	C10B—C11B—H11B	120.0
N1A—C7A—C6A	127.4 (5)	C12B—C11B—H11B	120.0
N1A—C7A—H7AA	116.3	C13B—C12B—C11B	120.0
C6A—C7A—H7AA	116.3	C13B—C12B—H12B	120.0
N1A—C8A—C9A	112.8 (5)	C11B—C12B—H12B	120.0
N1A—C8A—H8AA	109.0	C12B—C13B—C14B	120.0
C9A—C8A—H8AA	109.0	C12B—C13B—H13B	120.0
N1A—C8A—H8AB	109.0	C14B—C13B—H13B	120.0
C9A—C8A—H8AB	109.0	C13B—C14B—N2B	120.0
H8AA—C8A—H8AB	107.8	C13B—C14B—H14B	120.0
C10A—C9A—C8A	114.2 (5)	N2B—C14B—H14B	120.0
C10A—C9A—H9AA	108.7	C9C—C8C—N1B	117 (3)
C8A—C9A—H9AA	108.7	C9C—C8C—H8CA	107.9
C10A—C9A—H9AB	108.7	N1B—C8C—H8CA	107.9
C8A—C9A—H9AB	108.7	C9C—C8C—H8CB	107.9
H9AA—C9A—H9AB	107.6	N1B—C8C—H8CB	107.9
N2A—C10A—C11A	120.0 (6)	H8CA—C8C—H8CB	107.2
N2A—C10A—C9A	119.0 (5)	C10C—C9C—C8C	115.6 (11)
C11A—C10A—C9A	121.1 (6)	C10C—C9C—H9CA	108.4
C12A—C11A—C10A	121.0 (7)	C8C—C9C—H9CA	108.4
C12A—C11A—H11A	119.5	C10C—C9C—H9CB	108.4
C10A—C11A—H11A	119.5	C8C—C9C—H9CB	108.4
C13A—C12A—C11A	118.4 (7)	H9CA—C9C—H9CB	107.4
C13A—C12A—H12A	120.8	C10C—N2C—C14C	120.0
C11A—C12A—H12A	120.8	C10C—N2C—Ni2	122.7 (9)
C12A—C13A—C14A	118.8 (7)	C14C—N2C—Ni2	116.6 (9)
C12A—C13A—H13A	120.6	C11C—C10C—N2C	120.0
C14A—C13A—H13A	120.6	C11C—C10C—C9C	120.5 (7)
N2A—C14A—C13A	123.5 (7)	N2C—C10C—C9C	119.4 (7)
N2A—C14A—H14A	118.2	C10C—C11C—C12C	120.0
C13A—C14A—H14A	118.2	C10C—C11C—H11C	120.0
N1B—Ni2—N1B^i^	165.8 (3)	C12C—C11C—H11C	120.0
N1B—Ni2—O1B^i^	85.91 (17)	C13C—C12C—C11C	120.0
N1B^i^—Ni2—O1B^i^	84.10 (18)	C13C—C12C—H12C	120.0
N1B—Ni2—O1B	84.10 (18)	C11C—C12C—H12C	120.0
N1B^i^—Ni2—O1B	85.91 (17)	C12C—C13C—C14C	120.0
O1B^i^—Ni2—O1B	90.8 (2)	C12C—C13C—H13C	120.0
N1B—Ni2—N2C	95.7 (6)	C14C—C13C—H13C	120.0
N1B^i^—Ni2—N2C	94.0 (6)	C13C—C14C—N2C	120.0
O1B^i^—Ni2—N2C	87.7 (7)	C13C—C14C—H14C	120.0
O1B—Ni2—N2C	178.5 (7)	N2C—C14C—H14C	120.0
N1B—Ni2—N2C^i^	94.0 (6)	O14—Cl1—O11	111.7 (4)
N1B^i^—Ni2—N2C^i^	95.7 (6)	O13A—Cl1—O11A	110.6 (4)
O1B^i^—Ni2—N2C^i^	178.5 (7)	O13A—Cl1—O12A	110.8 (4)
O1B—Ni2—N2C^i^	87.7 (7)	O11A—Cl1—O12A	110.2 (4)
N2C—Ni2—N2C^i^	93.8 (14)	O14—Cl1—O12	110.6 (4)
N1B—Ni2—N2B^i^	99.0 (2)	O11—Cl1—O12	109.9 (4)
N1B^i^—Ni2—N2B^i^	91.2 (2)	O13A—Cl1—O14A	109.1 (4)
O1B^i^—Ni2—N2B^i^	175.1 (2)	O11A—Cl1—O14A	108.1 (4)
O1B—Ni2—N2B^i^	90.2 (2)	O12A—Cl1—O14A	107.8 (4)
N1B—Ni2—N2B	91.2 (2)	O14—Cl1—O13	108.8 (4)
N1B^i^—Ni2—N2B	99.0 (2)	O11—Cl1—O13	108.0 (4)
O1B^i^—Ni2—N2B	90.2 (2)	O12—Cl1—O13	107.7 (4)
O1B—Ni2—N2B	175.1 (2)	C1S—O1S—H1S	109.5
N2B^i^—Ni2—N2B	89.3 (4)	O1S—C1S—H1S1	109.5
C1B—O1B—Ni2	124.3 (3)	O1S—C1S—H1S2	109.5
C7B—N1B—C8B	114.7 (6)	H1S1—C1S—H1S2	109.5
C7B—N1B—C8C	119.4 (10)	O1S—C1S—H1S3	109.5
C7B—N1B—Ni2	127.1 (4)	H1S1—C1S—H1S3	109.5
C8B—N1B—Ni2	117.9 (6)	H1S2—C1S—H1S3	109.5
			
Ni1—O1A—C1A—C2A	−139.6 (5)	O1B—C1B—C6B—C5B	178.3 (5)
Ni1—O1A—C1A—C6A	41.1 (7)	C2B—C1B—C6B—C5B	−0.8 (9)
O1A—C1A—C2A—C3A	−177.4 (6)	O1B—C1B—C6B—C7B	−1.6 (9)
C6A—C1A—C2A—C3A	2.0 (9)	C2B—C1B—C6B—C7B	179.3 (6)
C1A—C2A—C3A—C4A	−0.6 (10)	C4B—C5B—C6B—C1B	1.3 (9)
C2A—C3A—C4A—C5A	−1.5 (10)	C4B—C5B—C6B—C7B	−178.8 (6)
C2A—C3A—C4A—Br1	177.8 (5)	C8B—N1B—C7B—C6B	178.0 (8)
C3A—C4A—C5A—C6A	2.1 (10)	C8C—N1B—C7B—C6B	−168 (2)
Br1—C4A—C5A—C6A	−177.2 (4)	Ni2—N1B—C7B—C6B	5.5 (10)
O1A—C1A—C6A—C5A	178.0 (5)	C1B—C6B—C7B—N1B	−22.5 (10)
C2A—C1A—C6A—C5A	−1.4 (8)	C5B—C6B—C7B—N1B	157.6 (6)
O1A—C1A—C6A—C7A	−1.6 (8)	C7B—N1B—C8B—C9B	122.8 (8)
C2A—C1A—C6A—C7A	179.0 (6)	Ni2—N1B—C8B—C9B	−63.9 (10)
C4A—C5A—C6A—C1A	−0.6 (9)	N1B—C8B—C9B—C10B	77.1 (10)
C4A—C5A—C6A—C7A	179.1 (6)	C14B—N2B—C10B—C11B	0.0
C8A—N1A—C7A—C6A	−177.0 (6)	Ni2—N2B—C10B—C11B	−173.8 (6)
Ni1—N1A—C7A—C6A	−2.9 (9)	C14B—N2B—C10B—C9B	−172.0 (8)
C1A—C6A—C7A—N1A	−19.0 (9)	Ni2—N2B—C10B—C9B	14.3 (8)
C5A—C6A—C7A—N1A	161.4 (6)	C8B—C9B—C10B—C11B	137.7 (8)
C7A—N1A—C8A—C9A	−160.9 (6)	C8B—C9B—C10B—N2B	−50.3 (11)
Ni1—N1A—C8A—C9A	24.7 (7)	N2B—C10B—C11B—C12B	0.0
N1A—C8A—C9A—C10A	−70.4 (7)	C9B—C10B—C11B—C12B	172.0 (8)
C14A—N2A—C10A—C11A	1.4 (8)	C10B—C11B—C12B—C13B	0.0
Ni1—N2A—C10A—C11A	−174.7 (5)	C11B—C12B—C13B—C14B	0.0
C14A—N2A—C10A—C9A	−178.1 (5)	C12B—C13B—C14B—N2B	0.0
Ni1—N2A—C10A—C9A	5.7 (7)	C10B—N2B—C14B—C13B	0.0
C8A—C9A—C10A—N2A	54.1 (7)	Ni2—N2B—C14B—C13B	174.3 (5)
C8A—C9A—C10A—C11A	−125.5 (6)	C7B—N1B—C8C—C9C	−154.4 (15)
N2A—C10A—C11A—C12A	−1.3 (10)	Ni2—N1B—C8C—C9C	32 (3)
C9A—C10A—C11A—C12A	178.2 (6)	N1B—C8C—C9C—C10C	−71 (3)
C10A—C11A—C12A—C13A	0.4 (11)	C14C—N2C—C10C—C11C	0.0
C11A—C12A—C13A—C14A	0.5 (11)	Ni2—N2C—C10C—C11C	−169.7 (19)
C10A—N2A—C14A—C13A	−0.6 (9)	C14C—N2C—C10C—C9C	176 (2)
Ni1—N2A—C14A—C13A	175.6 (5)	Ni2—N2C—C10C—C9C	7 (3)
C12A—C13A—C14A—N2A	−0.4 (10)	C8C—C9C—C10C—C11C	−135 (3)
Ni2—O1B—C1B—C2B	−141.8 (5)	C8C—C9C—C10C—N2C	48 (4)
Ni2—O1B—C1B—C6B	39.1 (7)	N2C—C10C—C11C—C12C	0.0
O1B—C1B—C2B—C3B	−179.4 (6)	C9C—C10C—C11C—C12C	−176 (2)
C6B—C1B—C2B—C3B	−0.2 (10)	C10C—C11C—C12C—C13C	0.0
C1B—C2B—C3B—C4B	0.9 (11)	C11C—C12C—C13C—C14C	0.0
C2B—C3B—C4B—C5B	−0.5 (11)	C12C—C13C—C14C—N2C	0.0
C2B—C3B—C4B—Br2	−177.7 (5)	C10C—N2C—C14C—C13C	0.0
C3B—C4B—C5B—C6B	−0.6 (10)	Ni2—N2C—C14C—C13C	170.3 (18)
Br2—C4B—C5B—C6B	176.6 (5)		

Symmetry code: (i) −*x*+1, *y*, −*z*+1/2.

### Hydrogen-bond geometry (Å, º)

**Table:**

*D*—H···*A*	*D*—H	H···*A*	*D*···*A*	*D*—H···*A*
O1*A*—H1*A*···O1*B*	0.82 (2)	1.64 (3)	2.430 (5)	161 (8)
C7*A*—H7*AA*···O14	0.93	2.47	2.990 (9)	115
C9*A*—H9*AA*···O1*A*^i^	0.97	2.40	3.105 (7)	129
C9*A*—H9*AB*···O14^ii^	0.97	2.55	3.482 (12)	162
C11*A*—H11*A*···O14*A*^ii^	0.93	2.60	3.406 (12)	145
C14*A*—H14*A*···N1*A*^i^	0.93	2.67	3.126 (8)	111
C7*B*—H7*BA*···O11*A*^iii^	0.93	2.54	3.069 (14)	117
C9*B*—H9*BB*···Br1^iii^	0.97	3.12	3.859 (10)	134
C14*B*—H14*B*···N1*B*^i^	0.93	2.54	3.155 (9)	124
C9*C*—H9*CA*···O1*B*^i^	0.97	2.37	3.02 (3)	124
C13*C*—H13*C*···Br1^iv^	0.93	3.08	3.55 (2)	114
C14*C*—H14*C*···Br1^iv^	0.93	3.05	3.54 (2)	115
O1*S*—H1*S*···O12	0.82	2.12	2.907 (15)	162
O1*S*—H1*S*···O13	0.82	2.57	3.249 (15)	140
O1*S*—H1*S*···O13*A*	0.82	1.64	2.436 (16)	162
C1*S*—H1*S*3···O13	0.96	2.55	3.276 (19)	133

Symmetry codes: (i) −*x*+1, *y*, −*z*+1/2; (ii) −*x*+1, −*y*+1, −*z*; (iii) *x*−1/2, *y*+1/2, −*z*+1/2; (iv) −*x*+3/2, *y*+1/2, *z*.
